# CentroFinder: a multi-feature framework for *de novo* prediction of fungal regional centromeres

**DOI:** 10.1093/bioadv/vbag270

**Published:** 2026-09-12

**Authors:** Sahar Salimi, Sharon Colson, Michael Renfro, Li-Jun Ma, Mostafa Rahnama

**Affiliations:** School of Environmental Studies, Tennessee Technological University, Cookeville, TN 38505, United States; Information Technology Services, Tennessee Technological University, Cookeville, TN 38505, United States; Information Technology Services, Tennessee Technological University, Cookeville, TN 38505, United States; Department of Biochemistry and Molecular Biology, University of Massachusetts Amherst, Amherst, MA 01003, United States; School of Environmental Studies, Tennessee Technological University, Cookeville, TN 38505, United States; Department of Biology, Tennessee Technological University, Cookeville, TN 38505, United States

## Abstract

**Motivation:**

Centromeres are essential chromosomal loci, yet their computational identification remains challenging due to rapid sequence evolution, high repeat content, and the absence of conserved defining motifs. This challenge is particularly pronounced in fungi, where centromere architectures vary widely in size, sequence composition, and chromatin organization, limiting the effectiveness of single-feature or motif-based prediction approaches.

**Results:**

We present CentroFinder, a fungal-specific computational framework for *de novo* centromere prediction from long-read sequencing–based genome assemblies. CentroFinder integrates multiple genomic and long-read–derived features into a weighted scoring model to identify loci where centromere-associated signals converge. Benchmarking against experimentally mapped centromeres in *Cryptococcus deuterogattii*, *Magnaporthe oryzae*, and *Neurospora crassa* showed that 27 of 28 predicted intervals overlapped the corresponding experimental domains, yielding 96.4% chromosome-level detection sensitivity. Application to 11 additional fungal genomes produced one contiguous predicted centromeric region per chromosome, supporting the transferability of the workflow for chromosome-level centromere prediction.

**Availability and implementation:**

CentroFinder is freely available as open-source software at https://github.com/RahnamaLab/CentroFinder. The pipeline is designed for high-performance computing environments and leverages features derived from long-read sequencing data.

## 1 Introduction

Centromeres are indispensable chromosomal loci that orchestrate kinetochore assembly and enable the attachment of spindle microtubules, ensuring the equal segregation of sister chromatids during both mitosis and meiosis (Karpen and Allshire 1997, McKinley and Cheeseman 2016). Their essential role in maintaining genome stability is underscored by the fact that centromere malfunction is associated with aneuploidy, cancer, and developmental disorders (Jansen *et al.* 2007, Carroll *et al.* 2010, Saha *et al.* 2019). Despite this deeply conserved function, the underlying DNA sequences of centromeres exhibit extraordinary diversity and rapid evolution—an observation described as the “centromere paradox” (Henikoff *et al.* 2001, Malik and Henikoff 2009, Narayanan *et al.* 2024). As a result, centromere identity is governed not by a universal sequence motif but by the interplay among DNA sequence, repetitive content, chromatin organization, and epigenetic regulation (McKinley and Cheeseman 2016).

Centromeric and pericentromeric regions have long been among the most poorly resolved parts of eukaryotic genomes because their high repeat content hinders assembly and read mapping with short-read data (Miga 2015, 2020). The advent of long-read sequencing technologies such as PacBio HiFi and Oxford Nanopore, along with improved assembly algorithms, now enables complete reconstruction of these complex loci in many organisms (Rahnama *et al.* 2020, Cheng *et al.* 2021, Naish *et al.* 2021, Altemose *et al.* 2022), opening the door to systematic, sequence-based centromere analysis. The fungal kingdom exemplifies extreme centromere diversity, ranging from ≤400 bp genetically defined point centromeres in *Saccharomyces cerevisiae* to large regional centromeres exceeding 150 kb (Smith *et al.* 2012, Yadav *et al.* 2018a, Guin *et al.* 2020, Narayanan *et al.* 2024). Reliable identification of centromeres creates opportunities to distinguish centromeric repeats from other repeat-rich genomic compartments, improving the interpretation of chromosome-scale assemblies, detecting centromere repositioning, and relating chromosomal rearrangements to karyotype evolution, adaptation, and pathogenicity (Bracewell *et al.* 2019, Zhou *et al.* 2022). In fungal pathogens, where chromosome structure can change rapidly, centromere maps therefore provide a framework for asking how centromere organization influences genome stability and evolutionary potential (Yadav *et al.* 2018a, Narayanan *et al.* 2021, 2024). Because no single genomic feature universally defines centromeres across fungi—and even closely related species may vary drastically (Guin *et al.* 2020)—computational prediction remains challenging, particularly for large regional centromeres embedded in repetitive DNA.

Although several software tools exist for centromere prediction, none are specifically designed for fungi, and most rely on a single dominant feature such as DNA methylation or tandem repeats (Mastrorosa *et al.* 2024, Xu *et al.* 2024, Elphinstone *et al.* 2025). While such approaches may perform well in species with relatively uniform centromere architecture, they often achieve low recall in fungi because centromeres are highly heterogeneous and no single feature is universally present (Smith *et al.* 2012, Yadav *et al.* 2018a, Xu *et al.* 2024, Elphinstone *et al.* 2025). This gap highlights the need for a multi-feature, fungal-tailored prediction framework. Fungi represent a particularly important target group for such a framework because many fungal genomes, including those of non-model and pathogenic species, are now being assembled with long-read sequencing, but experimental centromere maps remain unavailable for most species. To address this challenge, we developed CentroFinder, a fungal-specific computational pipeline designed for *de novo* prediction of fungal regional centromeres that integrates multiple genomic and epigenetic signals to identify centromere locations in chromosome-level assemblies. By relying on sequence- and long-read–derived features rather than chromatin immunoprecipitation or CENP-A/CenH3 mapping, CentroFinder enables centromere prediction in newly sequenced fungal genomes and in species for which experimental centromere mapping is unavailable or infeasible. CentroFinder quantifies six centromere-oriented features—TE coverage, tandem-repeat density, CpG methylation deviation, gene depletion, GC depletion, and long-read depth anomalies—across the genome and combines them using a biologically informed weighted scoring model. By integrating these complementary signals and aggregating high-scoring regions into continuous intervals, CentroFinder provides a reproducible approach for predicting regional centromere candidates from fungal genome assemblies, supporting comparative analyses of karyotype evolution, centromere repositioning, and structural variation.

## 2 Methods

### 2.1 Genome assemblies and input data

All centromere predictions were performed using high-quality fungal genome assemblies generated from long-read sequencing technologies (PacBio HiFi or Oxford Nanopore). Because centromere identification depends strongly on assembly continuity, analyses were restricted to assemblies with long contiguous internal regions spanning centromeric loci, regardless of whether they were fully chromosome-level or partially contiguous. For genomes lacking published annotations, gene models were generated *de novo* using FunGAP (Min *et al.* 2017) to ensure standardized annotation quality across species.

The availability of fungal genomes meeting these requirements is increasing rapidly as PacBio HiFi and Oxford Nanopore sequencing are now routinely used for fungal genome assembly (Mondo and Grigoriev 2025). Many model, pathogenic, and population-level fungal datasets now include chromosome-scale or highly contiguous long-read assemblies, providing a growing set of use cases for CentroFinder. However, the method is most informative when centromeric regions are contained within single contigs or scaffolds and when raw long-read data are available for coverage and methylation profiling. Therefore, highly fragmented short-read assemblies, or assemblies lacking corresponding raw long-read data, may provide insufficient information for reliable centromere prediction.

### 2.2 Feature extraction for centromere prediction

To identify centromeres, we quantified a set of sequence- and long-read–derived genomic features known to characterize fungal regional centromeres, including transposable element (TE) density, tandem repeat (TR) density, DNA methylation signatures, GC content, gene density, and long-read sequencing coverage. All features were calculated in non-overlapping 1 kb windows across each chromosome and used as inputs for the integrated centromere-scoring model. Non-overlapping windows were retained to avoid generating highly redundant adjacent observations and because downstream candidate selection integrates scores across multiple neighboring windows. Thus, the final prediction is determined from an extended regional signal rather than from an isolated 1 kb segment. The 1 kb window was selected as the default based on a window-size sensitivity analysis across the three experimentally benchmarked genomes, as described in Section 2.4 and Table S4, available as supplementary data at *Bioinformatics Advances* online. Window length remains user configurable. CentroFinder is designed to identify fungal regional centromeres and is not specifically optimized for short, genetically defined point centromeres.

For interval-based features, TE density and TR density were quantified as the fraction of each 1 kb window physically covered by annotated transposable element (TE) or tandem repeat (TR) intervals, respectively (i.e. a coverage ratio ranging from 0.0 to 1.0), rather than as the number of annotated features. In contrast, gene density was quantified as the absolute number of annotated genes overlapping each window. Epigenetic and sequence-derived features—including DNA methylation (mean CpG methylation frequency), GC content, and long-read sequencing coverage—were summarized as continuous window-level values before min–max normalization, with long-read coverage further converted into a depth-anomaly metric relative to chromosome-wide median coverage.

#### 2.2.1 Transposable element annotation

TEs were annotated using Extensive *De novo* TE Annotator (EDTA) with default parameters (Su *et al.* 2021). Coding sequences were removed prior to TE annotation to avoid misclassification of protein-coding regions. The resulting GFF3 annotations were converted to BED format and summarized to compute TE density as the fraction of each window covered by TE annotations along each chromosome.

#### 2.2.2 Tandem repeat detection

TRs were identified using Tandem Repeats Finder (TRF) (Benson 1999). TRF outputs were converted to BED format using trf2bed, and TR coverage was aggregated to generate tandem-repeat density profiles defined as the fraction of each window covered by tandem-repeat intervals across chromosomes.

#### 2.2.3 DNA methylation detection from long-read sequencing

For genomes sequenced with PacBio HiFi, CpG methylation was computed using the ccsmeth workflow (Ni *et al.* 2023). First, PacBio subreads were converted into CCS (HiFi) reads using *pbccs* as implemented in ccsmeth. CCS reads were then aligned to the genome using *pbmm2* (https://github.com/PacificBiosciences/pbmm2). CpG methylation (5mC) was detected using ccsmeth call_mods with the *attbigru2s_b21.v3* model, and window-level methylation frequencies were computed with ccsmeth call_freqb in aggregate mode as mean CpG methylation frequency per 1 kb window. Resulting methylation-frequency tracks were exported in BED format for downstream integration.

For genomes sequenced with Oxford Nanopore, methylation was extracted from raw signal–derived base-modification tags embedded in aligned reads. Nanopore reads were aligned to each genome using a minimap2-based workflow, and the resulting BAM files were sorted and indexed (Li 2018). Base-resolution 5mC calls were obtained by converting the BAM files into BED format using modbam2bed (https://github.com/epi2me-labs/modbam2bed), which parses modified-base tags from individual reads. The resulting BED tracks provided per-site methylation information that was subsequently summarized as mean CpG methylation frequency in the 1 kb analysis windows used for feature extraction.

Although PacBio HiFi and Oxford Nanopore platforms rely on distinct sequencing chemistries and methylation-calling algorithms, both approaches yield quantitative CpG methylation signals that are comparable at the genomic window scale used in this study. CentroFinder converts mean window-level CpG methylation frequency into its absolute deviation from the chromosome-wide median before normalization. The resulting feature therefore captures local hypermethylation or hypomethylation relative to the chromosome background rather than absolute methylation frequency. In all cases, methylation tracks were summarized into uniform 1 kb windows prior to integration, ensuring consistency across sequencing technologies.

#### 2.2.4 Long-read coverage profiling

HiFi or Nanopore reads were aligned to each corresponding assembly using *pbmm2* (PacBio) or *minimap2* (Nanopore) (Li 2018). Per-base read depth was aggregated across the genome, and a depth-anomaly metric—defined as deviation from the chromosome-wide median coverage—was calculated to highlight regions enriched in structurally complex or low-mappability sequences.

#### 2.2.5 Gene-content and GC-content extraction

Gene coordinates from FunGap or published annotations were converted to BED format and intersected with genomic windows to compute gene counts, defined as the number of annotated genes overlapping each 1 kb window. GC content for each window was calculated directly from the genome assembly using *bedtools nuc* (Quinlan and Hall 2010) and represented the fraction of G + C bases within each 1 kb window. Low gene density (including windows with zero genes) and reduced GC content were interpreted as signatures of gene absence and GC depletion, respectively. These features were included because fungal centromeres typically exhibit low GC content and are depleted of protein-coding genes.

### 2.3 Centromere scoring and prediction

Each chromosome is partitioned into non-overlapping 1 kb windows, and the raw window-level measurements are converted into six centromere-oriented scoring features before integration. TE coverage and tandem-repeat coverage are min–max normalized directly. Gene depletion is calculated as 1 − the min–max-normalized gene count, and GC depletion is calculated as 1 − the min–max-normalized GC fraction, so that gene-poor and GC-poor windows receive higher feature values. CpG methylation deviation is calculated as the absolute difference between the mean methylation frequency in each window and the chromosome-wide median methylation frequency and is then min–max normalized. Long-read coverage anomaly is calculated analogously from the absolute deviation of local read coverage from the chromosome-wide median. All six transformed features are therefore scaled from 0 to 1 and are oriented so that larger values represent stronger centromere-associated evidence.

For each genomic window *j*, the Centromere Score is calculated as:


$$S_{j}=\sum_{i=1}^{6}w_{i}f_{ij}$$

where $S_{j}$ is the composite score for genomic window j, $f_{ij}$ is the normalized value of feature i in window j, and $w_{i}$ is the weight assigned to feature i. The index i represents the six incorporated features: tandem-repeat density, TE coverage, gene depletion, CpG methylation deviation, long-read coverage anomaly, and GC depletion.

Feature weights are specified as biologically informed heuristic values rather than coefficients estimated using a statistical or machine-learning model. The feature hierarchy is motivated by the prominent structural contribution of tandem repeats and transposable elements to many fungal regional centromeres, whereas gene depletion, CpG methylation deviation, long-read coverage anomaly, and GC depletion are treated as complementary supporting features. The 4/8/1/1/1/1 configuration, corresponding to tandem-repeat density/TE coverage/gene depletion/CpG methylation deviation/long-read coverage anomaly/GC depletion, was selected as the common starting configuration after comparative evaluation across the benchmark genomes.

All feature weights are exposed as user-adjustable parameters. However, one configuration is applied consistently across all chromosomes of a genome and is not adjusted independently for individual chromosomes. The recommended 4/8/1/1/1/1 configuration was applied without modification to *Cryptococcus deuterogattii* and *Neurospora crassa*. In *Magnaporthe oryzae*, increasing the GC-depletion weight from 1 to 2 across all chromosomes recovered the remaining experimentally defined centromere, resulting in a finalized genome-wide configuration of 4/8/1/1/1/2. No parameters were adjusted separately for individual chromosomes. Because feature values are normalized within each chromosome, the composite score is used only to rank candidate regions within that chromosome and should not be compared directly among chromosomes, genomes, or weight configurations. The weighted score represents the initial candidate-ranking stage; candidate regions are subsequently refined through chromosome-end exclusion, regional score accumulation, and restriction to continuous gene-free or strongly gene-depleted intervals.

To evaluate sensitivity to feature-weight choices across different fungal centromere architectures, CentroFinder was applied to all three experimentally benchmarked genomes using seven predefined balanced, repeat-enriched, and feature-reduced configurations while all non-weight parameters were held constant. Experimentally defined centromere coordinates were not used as feature inputs or during chromosome-level scoring or candidate selection; they were used to compare positional recovery under the alternative configurations. For each configuration, recovery was assessed as the number of benchmark chromosomes for which the predicted interval overlapped the corresponding experimentally defined centromere. The configuration-level summary is provided in Table S3A, available as supplementary data at *Bioinformatics Advances* online, and the corresponding chromosome-level predictions are provided in Tables S3B–H, available as supplementary data at *Bioinformatics Advances* online. The complete parameter-selection and sensitivity-evaluation procedure is provided in Supplementary Method S1, available as supplementary data at *Bioinformatics Advances* online.

To prioritize extended regional signals rather than isolated high-scoring windows, CentroFinder calculates a centered rolling sum of the individual window scores. With the default 1 kb non-overlapping windows and an accumulation span of 50 windows, each accumulated score summarizes centromere-associated evidence across an approximately 50 kb neighborhood. Windows within chromosome-end exclusion zones are removed before candidate selection to reduce the influence of repeat-rich, gene-poor terminal regions. The default configuration excludes 100 kb from each end of chromosomes at least 500 kb long and 10 kb from each end of shorter sequences. These exclusion distances and the chromosome-length cutoff are user configurable. If the combined terminal exclusions leave no valid internal sequence, the chromosome is skipped with a warning. The window with the highest accumulated score is used as the initial anchor of the strongest internal regional signal. When this window overlaps an annotated gene, the anchor is moved to the nearest gene-free window, and the continuous gene-free island containing the adjusted anchor is identified. Within this island, the gene-free window with the highest accumulated score is designated as the final anchor. Windows with accumulated scores equal to or greater than 35% of the maximum accumulated score within the selected island are retained, and qualifying windows separated by no more than 2 kb are merged. The merged interval containing the final anchor is reported as the predicted centromeric region. Thus, the final prediction represents a multi-window regional interval supported by elevated accumulated scores rather than the single 1 kb window with the highest individual feature score. Because candidate intervals are assembled from non-overlapping genomic windows, their coordinate granularity is determined by the selected window size. With the default 1 kb windows, predicted start and end coordinates occur at 1 kb increments. These coordinates should therefore be interpreted as window-level estimates of regional centromere boundaries rather than base-pair-resolution boundary definitions.

### 2.4 Benchmarking using genomes with experimentally validated centromeres

Pipeline performance was evaluated using a curated benchmarking dataset consisting of three fungal species with experimentally mapped centromeres [*C. deuterogattii* R265 (Yadav *et al.* 2018b), *M. oryzae* Guy11 (Yadav *et al.* 2019), and *N. crassa* FGSC2489 OR74A (Smith *et al.* 2011)]. For each species, predicted centromeres were compared against published centromere coordinates, and accuracy metrics were computed per chromosome and per species. Because CentroFinder reports one candidate genomic interval per chromosome, performance was evaluated using interval-based positional metrics. For each prediction and experimentally defined centromere, interval precision was calculated as the fraction of the predicted interval overlapping the experimental domain, interval recall as the fraction of the experimental domain covered by the prediction, and intersection-over-union as the overlap divided by the combined genomic span of the two intervals. Midpoint distance was calculated as the absolute distance between the centers of the predicted and experimental intervals. Chromosome-level detection sensitivity was defined as the proportion of evaluated chromosomes for which the predicted interval overlapped the corresponding experimentally defined centromere. Because the experimentally defined centromere coordinates were used to compare predefined parameter configurations, the reported results represent calibrated benchmark performance rather than evaluation on an independent held-out dataset.

To provide a point of comparison with existing centromere-related tools, CDR-Finder (Mastrorosa *et al.* 2024), CentroMiner/QuarTeT (Lin *et al.* 2023), HiCAT (Gao *et al.* 2023), TRASH (Wlodzimierz *et al.* 2023), and StainedGlass (Vollger *et al.* 2022) were evaluated across the three benchmark fungal genomes using default or recommended settings, with additional parameter combinations evaluated where indicated. These tools were selected because they represent available approaches for centromere-dip region detection, centromere prediction, higher-order repeat detection, tandem repeat discovery, or repeat self-similarity visualization. Because several of these tools were developed for different centromere architectures or produce non-coordinate outputs, evaluation focused on whether each tool generated discrete candidate centromere coordinates suitable for comparison with experimentally defined centromeres. The output behavior of each comparator tool across the three benchmark fungal genomes was summarized in Table 2.

**Table 1:** Interval-based performance of CentroFinder against experimentally defined centromeres in three fungal genomes.^a^

Species	Chromosomes evaluated	Detection sensitivity	Prediction length range (kb)	EDC length range (kb)	Median interval precision	Median interval recall	Median IoU	Median midpoint distance (kb)
***C. deuterogattii***	14	13/14 (92.9%)	1.0–20.0	8.8–21.7	100%	90.1%	90.1%	0.24
***M. oryzae***	7	7/7 (100.0%)	56.0–108.0	57.3–108.7	100%	99.4%	99.4%	0.18
***N. crassa***	7	7/7 (100.0%)	18.0–152.0	315.7–423.2	100%	30.5%	30.5%	60.81
**Overall**	**28**	**27/28 (96.4%)**	**1.0–152.0**	**8.8–423.2**	**100%**	**90.1%**	**90.1%**	**0.33**

a Chromosomes evaluated include only chromosomes for which published experimentally defined centromere (EDC) coordinates were available. Detection sensitivity represents the number and percentage of evaluated chromosomes for which the CentroFinder-predicted interval overlapped the corresponding EDC under a strict interval-overlap criterion. Prediction length and EDC length are reported as minimum–maximum ranges. Interval precision was calculated as the overlap length divided by the predicted interval length, interval recall as the overlap length divided by the EDC length, and intersection-over-union (IoU) as the overlap length divided by the union of the predicted and experimental intervals. Midpoint distance represents the absolute distance between the centers of the predicted interval and the EDC. Precision, recall, IoU, and midpoint distance are reported as medians across the evaluated chromosomes for each species. For a non-overlapping prediction, interval precision, recall, and IoU were assigned values of zero. The overall row was calculated across all 28 chromosome-level comparisons rather than from the species-level summary values. See Table S1, available as supplementary data at *Bioinformatics Advances* online, for chromosome-level details. EDC coordinates were unavailable for *N. crassa* scaffolds 8 and 9; therefore, these scaffolds were not included in the performance calculations.

**Table 2:** Comparative assessment of CentroFinder and existing centromere-related tools across three experimentally benchmarked fungal genomes.^a^

Tool	Primary purpose	Main input requirements	Principal output	Single centromere candidate per chromosome?	Observed result across fungal genomes
**CentroFinder**	Prediction of regional fungal centromeres using integrated genomic and epigenomic features	Genome assembly and selected feature annotations or sequencing-derived feature tracks	Ranked candidate intervals and one selected candidate interval per chromosome	Yes	Consistently generated one internal candidate centromere interval per chromosome.
**CDR-Finder**	Identification of hypomethylated centromere-dip regions within sequence-resolved α-satellite centromeres	Genome assembly, methylation-tagged long-read BAM and predefined BED coordinates for the centromeric region	Coordinates and methylation profiles of hypomethylated regions within α-satellite arrays	No	The method requires α-satellite higher-order repeat arrays and a previously defined centromeric search region. Fungal centromeres generally lack the human/primate α-satellite architecture required by the algorithm; therefore, the method is not suitable for *de novo* fungal centromere prediction.
**CentroMiner/QuarTeT**	Identification of candidate centromeric regions based primarily on tandem-repeatmonomers	Genome assembly	One or more repeat-supported candidate regions, tandem-repeat summaries and diagnostic plots	No	Did not generate one unique, interpretable coordinate-defined centromere interval per chromosome suitable for comparison with the experimentally defined centromeres.
**HiCAT**	Annotation of centromeric tandem-repeat monomers and higher-order repeat structures	Genome sequence or a predefined candidate centromeric sequence	Monomer and higher-order repeat annotations and structural plots	No	No interpretable higher-order repeat structures characteristic of the architectures targeted by HiCAT were detected.
**TRASH**	*De novo* identification and structural annotation of tandem repeats	Genome assembly in FASTA format; optional repeat templates	Coordinate-level tandem-repeat loci, repeat families and higher-order repeat annotations	No	Did not generate one unique coordinate-defined centromeric interval per chromosome suitable for direct centromere benchmarking.
**StainedGlass**	Visualization of sequence self-similarity and repeat organization	Genome assembly or extracted genomic regions	Sequence-identity heatmaps	No	Generated sequence self-similarity maps but did not generate discrete coordinate-defined centromere predictions.

a CentroFinder, CDR-Finder, CentroMiner/QuarTeT, HiCAT, TRASH, and StainedGlass were evaluated across *C. deuterogattii* R265, *M. oryzae* Guy11, and *N. crassa* OR74A. Tools were run using default or recommended settings, with additional parameter combinations evaluated where indicated in Section 2. For each tool, the table summarizes its primary purpose, principal input requirements, output type, ability to generate a single coordinate-defined centromere candidate per chromosome, and observed output across the three genomes. Because the comparator tools were developed for different centromere architectures or for related repeat-annotation and visualization tasks, the comparison focused on whether their outputs could support chromosome-level fungal centromere prediction and interval-based comparison with experimentally defined centromeres.

To evaluate the effect of window length on centromere prediction, the three benchmark genomes were analyzed using non-overlapping windows of 100, 250, 500, 1000, 2000, 5000, and 10 000 bp. Within each genome, feature weights and all other analysis parameters were held constant so that only window length varied. Predictions generated using each window size were compared with the experimentally defined centromeres by determining whether the predicted interval overlapped the corresponding experimental region. Results of the window-size sensitivity analysis are summarized in Table S4, available as supplementary data at *Bioinformatics Advances* online.

To evaluate sensitivity to chromosome-end exclusion distance, *C. deuterogattii* was analyzed using exclusion distances of 0, 50, 100, 250, 500, 750, and 1000 kb while the 1 kb window size, 4/8/1/1/1/1 feature-weight configuration, and all other parameters were held constant. For each setting, we recorded whether each chromosome returned a prediction, whether the predicted interval overlapped or was fully contained within the corresponding experimentally defined centromere, and whether its coordinates were identical to those obtained with the 100 kb default. Results are summarized in Table S5, available as supplementary data at *Bioinformatics Advances* online.

### 2.5 Evaluation using additional fungal genomes

To evaluate the broader applicability and parameter transferability of CentroFinder beyond species with experimentally defined centromeres, the finalized workflow was applied to 11 additional fungal genomes comprising 96 chromosomes (Tables S2A and B, available as supplementary data at *Bioinformatics Advances* online). The recommended 4/8/1/1/1/1 feature-weight configuration and default chromosome-end exclusion parameters were applied first to every genome. This complete default parameter set was retained without modification in eight genomes. In *Aspergillus luchuensis* and *Penicillium psychrofluorescens*, diagnostic profiles containing unusually compact candidate intervals supported genome-wide increases in the GC-depletion weight from 1 to 5 and from 1 to 2, respectively. In *Fusarium oxysporum* f. sp. *vasinfectum* ME23, terminal regions with strong CpG methylation deviations produced competing subtelomeric signals, and the large-chromosome end-exclusion distance was therefore increased from 100 to 350 kb while retaining the 4/8/1/1/1/1 feature weights. Each adjustment was applied consistently across all chromosomes of the corresponding genome; no parameters were adjusted separately for individual chromosomes. Because experimentally mapped centromeres are unavailable for these species, evaluation focused on whether CentroFinder produced one contiguous predicted region per chromosome supported by concordance among multiple centromere-associated features. These regions remain candidates pending experimental validation.

## 3 Results

### 3.1 CentroFinder identifies regional centromeres in three benchmark fungal genomes

CentroFinder identified one predicted centromeric interval per chromosome across three benchmark fungal species with well-characterized regional centromeres (Smith *et al.* 2011, Yadav *et al.* 2018b, 2019). These species differ substantially in genome architecture, chromosome size, and repeat landscape, providing an evaluation across distinct fungal regional-centromere contexts (Table S1, available as supplementary data at *Bioinformatics Advances* online). Across the benchmark genomes, predicted intervals were supported by multiple regional-centromere-associated features, including GC depletion, transposable-element and tandem-repeat coverage, gene depletion, CpG methylation deviation, and long-read coverage anomalies (Fig. 1). These profiles represent the evidence used by CentroFinder for prediction; positional performance was assessed separately through comparison with CENP-A/CenH3-defined centromeres in Section 3.2.

**Figure 1:**
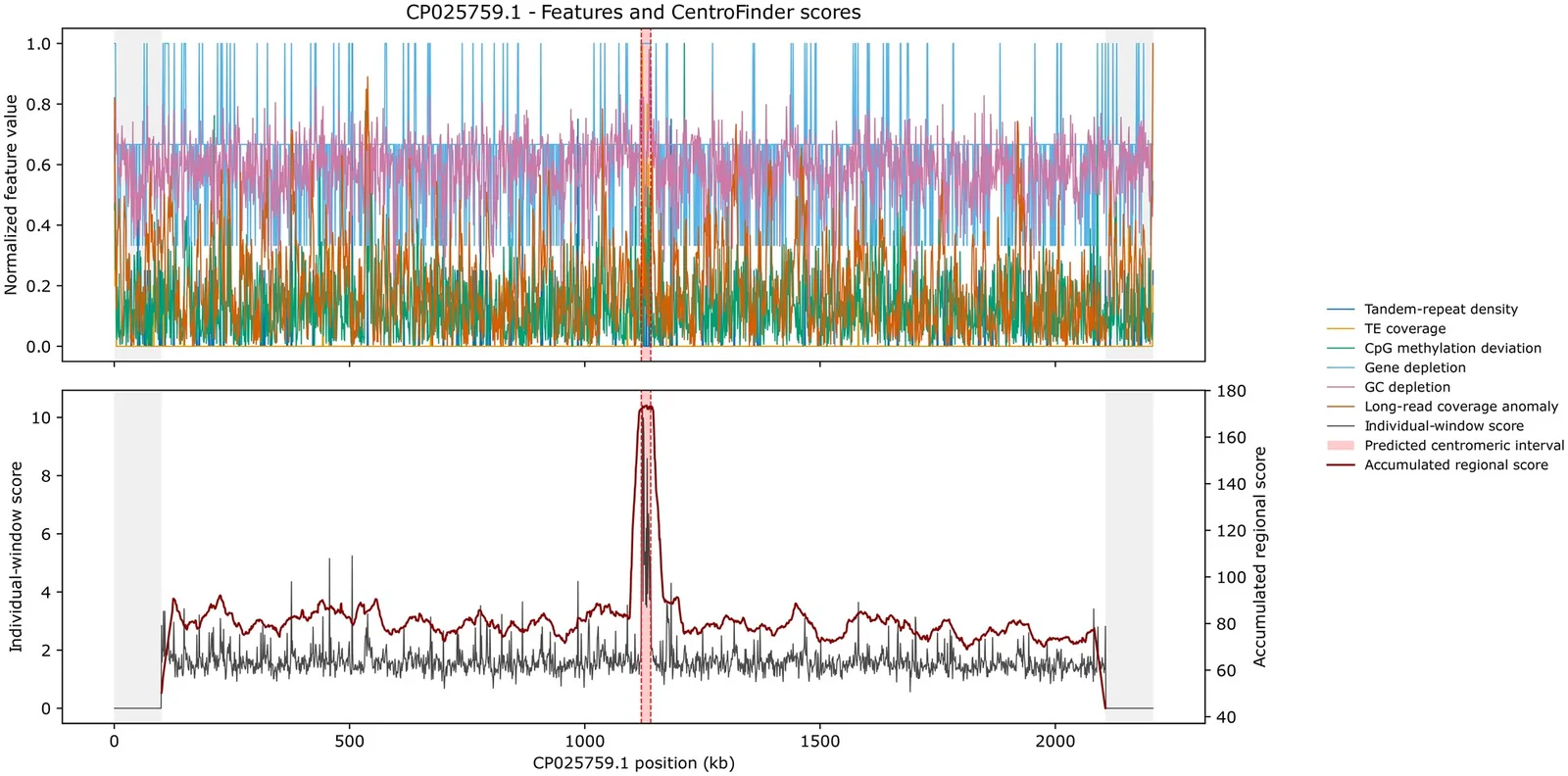
Representative CentroFinder output for chromosome CP025759.1 of *C. deuterogattii* R265. The upper panel shows the six normalized, centromere-oriented features calculated in non-overlapping 1 kb windows: tandem-repeat density, TE coverage, CpG methylation deviation, gene depletion, GC depletion, and long-read coverage anomaly. The lower panel shows the individual-window CentroFinder score in black on the left *y*-axis and the centered accumulated regional score calculated across 50 consecutive windows in dark red on the right *y*-axis. CentroFinder identifies the chromosome-wide maximum of the accumulated regional score and defines the final predicted centromeric interval within the corresponding continuous gene-free region. The predicted interval is indicated by pink shading and dashed red boundaries. Grey terminal regions represent the chromosome-end exclusion zones. Genomic position is shown in kilobases.

Across species, predicted centromere sizes varied in accordance with known centromere architectures. In *C. deuterogattii*, predicted intervals spanned approximately 1–20 kb, whereas larger intervals were observed in *M. oryzae* (approximately 56–108 kb) and *N. crassa* (approximately 18–152 kb) (Table S1, available as supplementary data at *Bioinformatics Advances* online). This pattern underscores the method’s ability not only to detect centromeres but also to prioritize compact centromeric candidate intervals within broader centromeric regions. Importantly, predictions were obtained using sequence- and long-read–derived features alone, without requiring chromatin immunoprecipitation or CENP-A mapping, demonstrating the tool’s utility for newly sequenced genomes or species where experimental mapping is not feasible.

### 3.2 High concordance between CentroFinder predictions and experimentally defined centromeres

To assess prediction accuracy, CentroFinder predictions were directly compared with experimentally defined centromeres (EDCs) derived from CENP-A or CenH3 mapping in three fungal species: *C. deuterogattii* R265, *M. oryzae* Guy11, and *N. crassa* OR74A. Across the 28 chromosomes with available experimentally defined centromeres, 27 CentroFinder predictions overlapped their corresponding experimental domains, yielding a chromosome-level detection sensitivity of 96.4%. Across all benchmark chromosomes, the median interval precision, interval recall, and intersection-over-union were 100%, 90.1%, and 90.1%, respectively, and the median midpoint distance was 0.33 kb (Table 1; Table S1, available as supplementary data at *Bioinformatics Advances* online). These complementary metrics distinguish correct localization of the predicted locus from differences in the genomic extent of the predicted and experimentally defined intervals. Chromosome-level precision, IoU, and EDC-overlap results are visualized in Fig. S1, available as supplementary data at *Bioinformatics Advances* online.

In *C. deuterogattii*, 13 of the 14 predicted intervals overlapped the corresponding experimentally defined centromeres. Twelve predictions were fully contained within the experimental domains, and one extended only 295 bp beyond an experimental boundary. The prediction on chromosome CP025765.1, however, spanned 394 000–395 000 bp, whereas the published EDC spanned 412 760–421 530 bp. The predicted interval was therefore separated from the EDC by approximately 17.8 kb and represents the single non-overlapping prediction under the strict interval-overlap criterion. Despite this exception, the species-level median interval precision, recall, and intersection-over-union were 100%, 90.1%, and 90.1%, respectively, and the median midpoint distance was 0.24 kb (Table 1; Table S1, available as supplementary data at *Bioinformatics Advances* online).

A similar pattern of strong positional concordance was observed in *M. oryzae*, where all seven CentroFinder predictions overlapped the experimentally mapped CenH3-associated domains (Table 1; Table S1, available as supplementary data at *Bioinformatics Advances* online). Predicted intervals spanned approximately 56–108 kb, compared with approximately 57–109 kb for the experimentally defined domains. The median interval precision was 100%, median interval recall was 99.4%, median intersection-over-union was 99.4%, and median midpoint distance was 0.18 kb, indicating close agreement in both genomic position and interval extent.

In *N. crassa*, all seven predictions with available experimental coordinates were fully contained within their corresponding experimentally defined domains, resulting in 100% interval precision. However, interval recall ranged from 5.7% to 40.6%, with a median of 30.5%, because CentroFinder predictions spanned approximately 18–152 kb whereas the experimentally defined domains spanned approximately 316–423 kb. The median midpoint distance was 60.8 kb. Thus, the large differences previously presented as positional offsets primarily reflect differences in interval extent: CentroFinder localized compact, feature-supported subregions within the broad CENP-A/CenH3-defined domains rather than selecting unrelated chromosomal loci (Table 1; Table S1, available as supplementary data at *Bioinformatics Advances* online).

Comparative analysis with existing centromere-related tools further highlighted the need for a fungal-tailored, coordinate-level prediction framework. CDR-Finder was not directly transferable because fungal centromeres lack the α-satellite higher-order repeat arrays used to define centromere-dip regions. CentroMiner/QuarTeT did not produce one unique, interpretable coordinate-defined centromere interval per chromosome suitable for comparison with the experimentally defined centromeres. HiCAT did not detect higher-order repeat structures, consistent with the absence of canonical Higher-Order Repeats (HOR) arrays in fungal regional centromeres. TRASH did not resolve a unique centromeric interval per chromosome, and StainedGlass generated repeat self-similarity maps for large repeat structures but did not produce discrete centromere coordinates suitable for quantitative interval comparison with experimentally defined centromeres (Table 2).

Cross-genome evaluation of the seven predefined feature-weight configurations identified 4/8/1/1/1/1 as the highest-performing common starting configuration. This configuration produced EDC-overlapping predictions on 26 of 28 benchmark chromosomes, including 13 of 14 in *C. deuterogattii*, 6 of 7 in *M. oryzae*, and 7 of 7 in *N. crassa*. The six alternative configurations recovered 19–22 of the 28 benchmark centromeres. Several alternative configurations performed well in one species but poorly in another, whereas 4/8/1/1/1/1 provided the highest overall recovery across the three benchmark genomes. These results support its use as the common starting configuration while also showing that prediction recovery varies with the relative feature weights (Table S3A; Tables S3B–H, available as supplementary data at *Bioinformatics Advances* online).

Window-size sensitivity analysis showed that 1 kb windows provided the highest overall recovery across the three benchmark genomes. EDC-overlapping predictions were recovered on 19, 20, 21, 26, 24, 24, and 18 of the 28 chromosomes using window sizes of 100, 250, 500, 1000, 2000, 5000, and 10 000 bp, respectively. The 1 kb window was therefore retained as the recommended default because it provided the highest overall recovery while maintaining kilobase-scale localization of regional centromeres (Table S4, available as supplementary data at *Bioinformatics Advances* online).

Chromosome-end exclusion sensitivity analysis in *C. deuterogattii* showed that exclusion distances of 50 and 100 kb produced the highest EDC overlap, with 13 of 14 predictions overlapping the corresponding experimental domains and all 14 chromosomes returning predictions. Without chromosome-end exclusion, overlap decreased to 11 of 14 chromosomes. At 250 kb, overlap declined to 8 of 14, and at exclusion distances of 500 kb or greater, progressively fewer chromosomes retained sufficient internal sequence for prediction. The 100 kb distance was therefore retained as the default for chromosomes at least 500 kb long because it provided the same maximal recovery as 50 kb while applying a wider terminal buffer without reducing the number of evaluable chromosomes (Table S5, available as supplementary data at *Bioinformatics Advances* online).

Together, these benchmarking results show strong positional agreement in the three evaluated fungal species. CentroFinder generated benchmarkable coordinate-defined predictions for all 28 evaluable chromosomes, whereas CentroMiner/QuarTeT, HiCAT, TRASH, and StainedGlass generated 0/28 equivalent benchmarkable predictions; CDR-Finder was not applicable.

### 3.3 Integrated multi-feature scoring delineates predicted centromeric regions

Across the benchmark genomes, each individual centromere-associated feature—including GC depletion, transposable-element and tandem-repeat coverage, gene depletion, CpG methylation deviation, and long-read coverage anomaly—frequently marked multiple genomic regions, particularly in repeat-rich or structurally complex backgrounds, and was therefore insufficient on its own to identify a unique centromeric locus. In contrast, integrating these features into a composite centromere score resulted in the convergence of multiple centromere-associated signals at a single internal locus per chromosome. This convergence produced a local cluster of elevated individual-window scores and the chromosome-wide maximum of the accumulated regional score, supporting selection of one candidate centromeric interval per chromosome (Fig. 1). This pattern was observed across the three benchmark species, which differ markedly in centromere size, repeat composition, and chromatin architecture (Table S1, available as supplementary data at *Bioinformatics Advances* online).

Feature integration was particularly informative in *N. crassa*, where individual features broadly marked extensive repeat-rich domains, whereas their combined signal identified compact predicted centromeric intervals within the larger experimentally defined regions (Table 1). In *M. oryzae*, integration reduced the influence of high-scoring non-centromeric signals in subtelomeric and repeat-dense regions, yielding one predicted centromeric interval per chromosome.

To further evaluate the broader applicability of CentroFinder, the finalized workflow was applied to 11 additional fungal genomes comprising 96 chromosomes (Tables S2A and B, available as supplementary data at *Bioinformatics Advances* online). The common 4/8/1/1/1/1 feature-weight configuration produced one contiguous predicted region per chromosome in all 11 genomes. The complete default parameter set was retained without modification in eight genomes. The common feature weights were retained in nine genomes; *A. luchuensis* and *P. psychrofluorescens* required genome-wide increases in the GC-depletion weight to 5 and 2, respectively. *F. oxysporum* f. sp. *vasinfectum* retained the common feature weights but required an increase in the large-chromosome end-exclusion distance from 100 to 350 kb to reduce the influence of terminal regions showing strong CpG methylation deviations. Each adjustment was applied consistently across all chromosomes of the corresponding genome. Because experimentally defined centromeres are unavailable for these genomes, these analyses demonstrate parameter transferability and the generation of coherent chromosome-level predictions rather than experimental prediction accuracy, and an absolute false-positive rate cannot be determined for these datasets.

Together, these results show that integrating complementary sequence- and long-read–derived features enabled CentroFinder to identify discrete centromeric regions in the three benchmark genomes and generate coherent chromosome-level predictions in the additional genomes. The experimentally benchmarked and additional-genome analyses support the utility and transferability of this feature-integration strategy across the fungal species evaluated here, while broader performance across fungal lineages will require further experimental validation.

## 4 Discussion

Accurate identification of centromeres remains a fundamental challenge in genome analysis due to their rapid sequence evolution, high repeat content, and lack of conserved defining motifs (Henikoff *et al.* 2001, Smith *et al.* 2011, Miga 2020). This challenge is particularly pronounced in fungi, where centromere architectures span an exceptional spectrum of sizes, sequence compositions, and epigenetic configurations even among closely related species (Roy and Sanyal 2011, Smith *et al.* 2012, Friedman and Freitag 2017, Guin *et al.* 2020). In this study, we introduce CentroFinder, a fungal-specific computational framework that integrates multiple genomic and long-read–derived features for *de novo* prediction of regional centromeres from genome assemblies.

A central insight underlying CentroFinder is that no single genomic signal is sufficient to reliably define fungal centromeres across lineages. Features such as repeat enrichment, GC depletion, gene absence, CpG methylation deviation, or read-depth irregularities can each occur elsewhere in the genome, particularly in subtelomeric or structurally dynamic regions. By explicitly integrating these heterogeneous signals within a biologically informed weighted scoring framework, CentroFinder identifies the unique internal locus on each chromosome where centromere-associated features converge most strongly. In the evaluated genomes, this integrative strategy distinguished predicted centromeric regions from other repetitive or compositionally biased loci.

The individual features incorporated into CentroFinder are established centromere-associated characteristics and are not themselves presented as novel. The methodological contribution of CentroFinder is a standardized and reproducible workflow that extracts and normalizes these features chromosome-wide, integrates them with long-read–derived signals, and predicts a discrete regional centromeric interval without requiring prior centromere coordinates or CENP-A/CenH3 mapping. Consistent with this design, feature-weight sensitivity analysis showed that prediction recovery remained substantial across alternative configurations, including configurations in which the gene-depletion weight was set to zero, although cross-genome recovery was lower than with the selected 4/8/1/1/1/1 configuration.

Quantitative performance was evaluated in three fungal species with experimentally defined regional centromeres (Smith *et al.* 2011, Yadav *et al.* 2018b, 2019). Across these species, CentroFinder predicted one centromeric region per chromosome, with 27 of 28 predictions overlapping the corresponding experimental domains. Application of the finalized workflow to 11 additional fungal genomes comprising 96 chromosomes produced one contiguous predicted centromeric region per chromosome. The common 4/8/1/1/1/1 feature weights were retained in nine genomes, one of which required a larger chromosome-end exclusion distance; the remaining two genomes required genome-wide GC-depletion adjustments. Because experimentally defined centromeres are unavailable for these additional genomes, these analyses support the transferability of the workflow and its ability to generate coherent chromosome-level predictions but do not independently establish prediction accuracy in those species or universal performance across fungi. The integration strategy does not require predefined repeat families or species-specific sequence motifs, which may facilitate its application across fungal regional-centromere architectures while still requiring genome-specific evaluation.

Interval-based benchmarking revealed two distinct patterns of agreement with experimentally defined centromeres. In *M. oryzae* and most *C. deuterogattii* chromosomes, CentroFinder predictions closely matched the positions and genomic extents of the experimental domains, resulting in high interval precision, recall, and intersection-over-union. In *N. crassa*, in contrast, all predictions were fully contained within the experimental domains but showed substantially lower recall because the CENP-A/CenH3-defined domains were much broader than the compact intervals prioritized by CentroFinder. Chromatin-based mapping using CENP-A or CenH3 ChIP-seq can identify extended enrichment domains that include both centromeric and surrounding pericentromeric chromatin, particularly in species with large regional centromeres (Smith *et al.* 2011, Bodor *et al.* 2014, Altemose *et al.* 2022, Naish and Henderson 2024). The *N. crassa* results therefore indicate that the integrated sequence- and long-read–derived features converge most strongly within a subregion of the broader chromatin domain rather than uniformly across its complete extent.

Chromosome CP025765.1 of *C. deuterogattii* illustrates a different limitation. The final predicted interval occurred approximately 17.8 kb upstream of, and did not overlap, the published experimental domain. Under the strict interval-overlap criterion, this prediction represents a false localization, although it remained in the adjacent genomic neighborhood. The unusually compact 1 kb prediction suggests that the sequence- and long-read–derived feature landscape on this chromosome did not coincide with the published CENP-A-defined domain under the finalized default parameters. This discrepancy could reflect local discordance between the sequence- and long-read–derived feature landscape and the chromatin-defined centromeric domain or chromosome-specific centromere architecture; the available data do not distinguish among these possibilities. This case emphasizes that CentroFinder prioritizes feature-supported candidate regions and that experimental centromere mapping remains necessary for definitive confirmation.

CentroFinder was intentionally developed as a fungal-specific framework rather than a general-purpose eukaryotic centromere predictor. Its scoring model incorporates genomic and epigenetic characteristics commonly associated with fungal regional centromeres. Accordingly, the default 1 kb window is appropriate for the regional centromeres targeted by CentroFinder but is not intended to resolve short, genetically defined point centromeres, such as the approximately 125 bp centromeres of *S. cerevisiae*. Consistent with this intended scope, application of CentroFinder to the *S. cerevisiae* genome did not accurately recover the known point centromeres. The use of non-overlapping 1 kb windows also constrains reported boundary coordinates to 1 kb increments. Although overlapping windows could provide finer coordinate increments, adjacent overlapping windows would contain largely redundant sequence and feature information and would not independently establish the underlying biological centromere boundaries. CentroFinder predictions should therefore be interpreted as regional candidate intervals rather than base-pair-resolution centromere coordinates. Because centromere architectures differ substantially among eukaryotic lineages, extending the methodology to additional taxonomic groups will require lineage-specific optimization of the feature-weighting framework and represents an important direction for future work.

CentroFinder may be applied to assemblies that are not fully complete, provided that the internal centromeric region is represented contiguously. In *M. oryzae*, the predicted intervals overlapped the corresponding CENP-A–defined domains even though the experimentally defined centromeres were originally mapped using a partially assembled genome. However, if a centromere is fragmented across multiple contigs or occurs at a contig boundary, the complete set of centromere-associated features may not be represented within a single sequence, potentially producing truncated predictions or preventing identification of a unique candidate centromeric region. The availability of sufficiently contiguous assemblies and corresponding raw long-read data therefore remains an important limitation, particularly in poorly sampled fungal lineages.

In summary, CentroFinder is an interpretable, biologically informed framework for *de novo* prediction of fungal regional-centromere candidates by integrating complementary genomic and long-read–derived signals. Quantitative benchmarking in three species showed 96.4% chromosome-level detection sensitivity, while application to 11 additional genomes demonstrated the generation of coherent candidate regions but not experimental prediction accuracy. Performance depends on assembly continuity, data quality, and the relevance of the selected features and parameters to the target genome. CentroFinder therefore provides a reproducible alternative to manual feature integration and can support hypothesis generation, prioritization of loci for experimental validation, and comparative studies of fungal centromere organization.

## Supplementary Material

vbag270_Supplementary_Data

## Data Availability

CentroFinder source code, configuration files, and documentation are openly available at https://github.com/RahnamaLab/CentroFinder. Publicly available genome assemblies and sequencing datasets used in this study are identified by accession numbers in Tables S1 and S2, available as supplementary data at *Bioinformatics Advances* online. All other data supporting the findings of this study are included in the article and its supplementary material.

## References

[vbag270-B1] Altemose N , Logsdon GA, Bzikadze AV et al Complete genomic and epigenetic maps of human centromeres. Science 2022;376:eabl4178. 10.1126/science.abl4178 PMC9233505 35357911

[vbag270-B2] Benson G. Tandem repeats finder: a program to analyze DNA sequences. Nucleic Acids Res 1999;27:573–80. 10.1093/nar/27.2.573 PMC148217 9862982

[vbag270-B3] Bodor DL , Mata JF, Sergeev M et al The quantitative architecture of centromeric chromatin. Elife 2014;3:e02137. 10.7554/eLife.02137 PMC4091408 25027692

[vbag270-B4] Bracewell R , Chatla K, Nalley MJ et al Dynamic turnover of centromeres drives karyotype evolution in Drosophila. Elife 2019;8:e49002. 10.7554/eLife.49002 PMC6795482 31524597

[vbag270-B5] Carroll CW , Milks KJ, Straight AF et al Dual recognition of CENP-A nucleosomes is required for centromere assembly. J Cell Biol 2010;189:1143–55. 10.1083/jcb.201001013 PMC2894454 20566683

[vbag270-B6] Cheng H , Concepcion GT, Feng X et al Haplotype-resolved *de novo* assembly using phased assembly graphs with hifiasm. Nat Methods 2021;18:170–5. 10.1038/s41592-020-01056-5 PMC7961889 33526886

[vbag270-B7] Elphinstone C , Elphinstone R, Todesco M et al RepeatOBserver: tandem repeat visualisation and putative centromere detection. Mol Ecol Resour 2025;25:e14084. 10.1111/1755-0998.14084 PMC12415947 40035343

[vbag270-B8] Friedman S , Freitag M. Centrochromatin of fungi. Prog Mol Subcell Biol 2017;56:85–109. 10.1007/978-3-319-58592-5_4 28840234

[vbag270-B9] Gao S , Yang X, Guo H et al HiCAT: a tool for automatic annotation of centromere structure. Genome Biol 2023;24:58. 10.1186/s13059-023-02900-5 PMC10053651 36978122

[vbag270-B10] Guin K , Sreekumar L, Sanyal K et al Implications of the evolutionary trajectory of centromeres in the fungal kingdom. Annu Rev Microbiol 2020;74:835–53. 10.1146/annurev-micro-011720-122512 32706633

[vbag270-B11] Henikoff S , Ahmad K, Malik HS et al The centromere paradox: stable inheritance with rapidly evolving DNA. Science 2001;293:1098–102. 10.1126/science.1062939 11498581

[vbag270-B12] Jansen LET , Black BE, Foltz DR et al Propagation of centromeric chromatin requires exit from mitosis. J Cell Biol 2007;176:795–805. 10.1083/jcb.200701066 PMC2064054 17339380

[vbag270-B13] Karpen GH , Allshire RC. The case for epigenetic effects on centromere identity and function. Trends Genet 1997;13:489–96. 10.1016/s0168-9525(97)01298-5 9433139

[vbag270-B14] Li H. Minimap2: pairwise alignment for nucleotide sequences. Bioinformatics 2018;34:3094–100. 10.1093/bioinformatics/bty191 PMC6137996 29750242

[vbag270-B15] Lin Y , Ye C, Li X et al quarTeT: a telomere-to-telomere toolkit for gap-free genome assembly and centromeric repeat identification. Hortic Res 2023;10:uhad127. 10.1093/hr/uhad127 PMC10407605 37560017

[vbag270-B16] Malik HS , Henikoff S. Major evolutionary transitions in centromere complexity. Cell 2009;138:1067–82. 10.1016/j.cell.2009.08.036 19766562

[vbag270-B17] Mastrorosa FK , Oshima KK, Rozanski AN et al Identification and annotation of centromeric hypomethylated regions with CDR-Finder. Bioinformatics 2024;40:btae733. 10.1093/bioinformatics/btae733 PMC11663805 39657946

[vbag270-B18] McKinley KL , Cheeseman IM. The molecular basis for centromere identity and function. Nat Rev Mol Cell Biol 2016;17:16–29. 10.1038/nrm.2015.5 PMC8603311 26601620

[vbag270-B19] Miga KH. Completing the human genome: the progress and challenge of satellite DNA assembly. Chromosome Res 2015;23:421–6. 10.1007/s10577-015-9488-2 26363799

[vbag270-B20] Miga KH. Centromere studies in the era of ‘telomere-to-telomere’ genomics. Exp Cell Res 2020;394:112127. 10.1016/j.yexcr.2020.112127 PMC8284601 32504677

[vbag270-B21] Min B , Grigoriev IV, Choi I-G et al FunGAP: fungal genome annotation pipeline using evidence-based gene model evaluation. Bioinformatics 2017;33:2936–7. 10.1093/bioinformatics/btx353 28582481

[vbag270-B22] Mondo SJ , Grigoriev IV. A genomic perspective on fungal diversity and evolution. Nat Rev Microbiol 2025;23:718–33. 10.1038/s41579-025-01195-6 40588585

[vbag270-B23] Naish M , Alonge M, Wlodzimierz P et al The genetic and epigenetic landscape of the arabidopsis centromeres. Science 2021;374:eabi7489. 10.1126/science.abi7489 PMC10164409 34762468

[vbag270-B24] Naish M , Henderson IR. The structure, function, and evolution of plant centromeres. Genome Res 2024;34:161–78. 10.1101/gr.278409.123 PMC10984392 38485193

[vbag270-B25] Narayanan A , Reza MH, Sanyal K et al Behind the scenes: centromere-driven genomic innovations in fungal pathogens. PLoS Pathog 2024;20:e1012080. 10.1371/journal.ppat.1012080 PMC10977804 38547101

[vbag270-B26] Narayanan A , Vadnala RN, Ganguly P et al Functional and comparative analysis of centromeres reveals clade-specific genome rearrangements in *Candida auris* and a chromosome number change in related species. mBio 2021;12:905. 10.1128/mBio.00905-21 PMC8262905 33975937

[vbag270-B27] Ni P , Nie F, Zhong Z et al DNA 5-methylcytosine detection and methylation phasing using PacBio circular consensus sequencing. Nat Commun 2023;14:4054. 10.1038/s41467-023-39784-9 PMC10329642 37422489

[vbag270-B28] Quinlan AR , Hall IM. BEDTools: a flexible suite of utilities for comparing genomic features. Bioinformatics 2010;26:841–2. 10.1093/bioinformatics/btq033 PMC2832824 20110278

[vbag270-B29] Rahnama M , Novikova O, Starnes JH et al Transposon-mediated telomere destabilization: a driver of genome evolution in the blast fungus. Nucleic Acids Res 2020;48:7197–217. 10.1093/nar/gkaa287 PMC7367193 32558886

[vbag270-B30] Roy B , Sanyal K. Diversity in requirement of genetic and epigenetic factors for centromere function in fungi. Eukaryot Cell 2011;10:1384–95. 10.1128/EC.05165-11 PMC3209047 21908596

[vbag270-B31] Saha AK , Mourad M, Kaplan MH et al The genomic landscape of centromeres in cancers. Sci Rep 2019;9:11259. 10.1038/s41598-019-47757-6 PMC6677800 31375789

[vbag270-B32] Smith KM , Galazka JM, Phatale PA et al Centromeres of filamentous fungi. Chromosome Res 2012;20:635–56. 10.1007/s10577-012-9290-3 PMC3409310 22752455

[vbag270-B33] Smith KM , Phatale PA, Sullivan CM et al Heterochromatin is required for normal distribution of *Neurospora crassa* CenH3. Mol Cell Biol 2011;31:2528–42. 10.1128/MCB.01285-10 PMC3133421 21505064

[vbag270-B34] Su W , Ou S, Hufford MB et al A tutorial of EDTA: extensive De novo TE annotator. Methods Mol Biol 2021;2250:55–67. 10.1007/978-1-0716-1134-0_4 33900591

[vbag270-B35] Vollger MR , Kerpedjiev P, Phillippy AM et al StainedGlass: interactive visualization of massive tandem repeat structures with identity heatmaps. Bioinformatics 2022;38:2049–51. 10.1093/bioinformatics/btac018 PMC8963321 35020798

[vbag270-B36] Wlodzimierz P , Hong M, Henderson IR et al TRASH: tandem repeat annotation and structural hierarchy. Bioinformatics 2023;39:btad308. 10.1093/bioinformatics/btad308 PMC10199239 37162382

[vbag270-B37] Xu D , Yang J, Wen H et al CentIER: accurate centromere identification for plant genomes. Plant Commun 2024;5:101046. 10.1016/j.xplc.2024.101046 PMC11573919 39118326

[vbag270-B38] Yadav V , Sreekumar L, Guin K et al Five pillars of centromeric chromatin in fungal pathogens. PLoS Pathog 2018a;14:e1007150. 10.1371/journal.ppat.1007150 PMC6107279 30138484

[vbag270-B39] Yadav V , Sun S, Billmyre RB et al RNAi is a critical determinant of centromere evolution in closely related fungi. Proc Natl Acad Sci USA 2018b;115:3108–13. 10.1073/pnas.1713725115 PMC5866544 29507212

[vbag270-B40] Yadav V , Yang F, Reza MH et al Cellular dynamics and genomic identity of centromeres in cereal blast fungus. mBio 2019;10:1581. 10.1128/mBio.01581-19 PMC6667624 31363034

[vbag270-B41] Zhou J , Liu Y, Guo X et al Centromeres: from chromosome biology to biotechnology applications and synthetic genomes in plants. Plant Biotechnol J 2022;20:2051–63. 10.1111/pbi.13875 PMC9616519 35722725

