# Supplementary material for: CentroFinder: a multi-feature framework for de novo prediction of fungal regional centromeres
Source: Bioinform Adv. 2026 Sep 12;6(1):vbag270. doi: 10.1093/bioadv/vbag270 (PMC13682844; doi:10.1093/bioadv/vbag270)
Supplement: Supplementary file 1 [file vbag270_supplementary_data.zip › Supplementary_Method_S1_FinalV3.docx]

**Supplementary Method S1.**

**Protocol for evaluating CentroFinder outputs and applying genome-wide parameter adjustments**

# S1.1 Purpose and general principle

CentroFinder should initially be run using the common default settings. The resulting chromosome-level diagnostic plots should then be inspected to determine whether the selected intervals are supported by coherent regional signals. Parameter adjustment is considered only when the default output shows a reproducible problem, such as an unusually narrow interval associated with an isolated score peak, no clear candidate region, multiple competing regional maxima, or recurrent selection of chromosome-terminal regions.

This evaluation is independent of whether experimentally defined centromere coordinates are available. When such coordinates exist, they may be used to assess positional agreement after prediction. However, the same plot-based evaluation and genome-wide adjustment procedure should be followed in both benchmark and non-benchmark genomes.

Parameter adjustments should not be made merely to force a prediction to match an expected centromere length or location. Instead, they should be based on the normalized feature profiles, the individual-window scores, the accumulated regional score, the gene-free structure of the selected region, and the behavior of the other chromosomes in the same genome.

**All adjustments should:**

**1.** begin from the common default configuration;

**2.** address a clearly identified problem in the diagnostic plots;

**3.** change only one parameter class at a time;

**4.** be applied genome-wide rather than to individual chromosomes;

**5.** be evaluated across all chromosome-level outputs; and

**6.** be retained only when they resolve the identified problem without destabilizing otherwise coherent predictions.

**The feature-weight order used throughout this protocol is:**

tandem-repeat density/TE coverage/gene depletion/CpG methylation deviation/long-read coverage anomaly/GC depletion.

# S1.2 Initial run using the common default settings

The initial CentroFinder analysis should use the following settings:

**Window size:** 1,000 bp

**Feature weights:**

Tandem-repeat density: 4

TE coverage: 8

Gene depletion: 1

CpG methylation deviation: 1

Long-read coverage anomaly: 1

GC depletion: 1

Common feature-weight configuration:

4/8/1/1/1/1

**Accumulated regional score:**

50 consecutive windows, corresponding to approximately 50 kb with the default 1-kb windows

**Large-sequence chromosome-end exclusion:**

100,000 bp

**Short-sequence chromosome-end exclusion:**

10,000 bp

**Short-sequence cutoff:**

500,000 bp

**Regional retention threshold:**

35% of the maximum accumulated score within the selected gene-free island

**Maximum gap between qualifying windows:**

2,000 bp

The 4/8/1/1/1/1 configuration should be applied to all chromosomes in the initial run. Different weights should not be assigned to individual chromosomes based on their repeat content, candidate length, or apparent score profile.

Before interpreting the results, confirm that all input tracks used in the selected configuration are present and correctly coordinated with the genome assembly. Chromosome or scaffold names must be consistent among the genome assembly and all included feature files. Missing feature tracks assigned non-zero weights, inconsistent sequence identifiers, assembly gaps, incomplete gene annotations, or collapsed repeat regions can generate anomalous score profiles that cannot be corrected through parameter adjustment.

# S1.3 Inspection of the chromosome-level diagnostic plots

The diagnostic plot for every chromosome should be examined after the initial run.

## S1.3.1 Feature profiles

**The upper panel displays the six normalized features used by CentroFinder:**

**•** tandem-repeat density;

**•** TE coverage;

**•** gene depletion;

**•** CpG methylation deviation;

**•** long-read coverage anomaly; and

**•** GC depletion.

Each feature is normalized within the chromosome. The profile therefore shows how strongly each window differs from the chromosome-wide background rather than providing an absolute comparison among different chromosomes or genomes.

## S1.3.2 Individual-window and accumulated regional scores

The black line in the lower panel represents the CentroFinder score calculated independently for each genomic window. Isolated high-scoring windows may be produced by individual transposable elements, tandem-repeat arrays, coverage artifacts, or other locally unusual sequence features. A single high black peak should therefore not automatically be interpreted as a centromeric region.

CentroFinder also calculates an accumulated regional score across 50 consecutive windows. When displayed, this score is shown as the dark red line. The accumulated score identifies regions in which elevated individual-window scores are clustered across a broader genomic interval.

The final candidate is anchored to the chromosome-wide maximum of the accumulated regional score within the searchable internal sequence. CentroFinder then evaluates the corresponding continuous gene-free region and defines the final candidate interval from the supported windows surrounding the regional maximum.

Consequently, the selected centromeric interval is expected to correspond to a regional cluster of elevated individual-window scores rather than necessarily containing the single highest individual-window score.

## S1.3.3 Characteristics of a coherent prediction

A coherent CentroFinder prediction generally has the following characteristics:

**•** one dominant internal accumulated-score maximum;

**•** a cluster of elevated individual-window scores rather than one isolated spike;

**•** location within a continuous gene-free or strongly gene-depleted region;

**•** support from several centromere-associated features;

**•** clear separation from chromosome-terminal repeat signals;

**•** no equally supported distant alternative region; and

**•** retention of the same general location under a limited, biologically reasonable parameter change.

Candidate length alone should not be used to accept or reject a prediction. Fungal regional centromeres can vary substantially in size. A short interval may be retained when it corresponds to a coherent regional maximum supported by multiple features. Conversely, a short interval should be examined more carefully when it represents only one isolated high-scoring window or when a broader competing internal region is visible.

# S1.4 Determining whether adjustment is required

The common settings should be retained when the chromosome-level plots show one dominant internal candidate supported by a coherent regional score and multiple centromere-associated features.

Adjustment should be considered when one or more of the following patterns occur:

**1.** no candidate interval is returned;

**2.** the selected interval is extremely narrow and associated with an isolated score spike;

**3.** several distant regions have similar accumulated regional scores;

**4.** the selected interval is dominated almost entirely by one feature;

**5.** a broad internal multi-feature region is visible but is outscored by a narrow isolated locus;

**6.** several chromosomes produce terminal or near-terminal candidates;

**7.** the selected candidate moves to a substantially different region after a small parameter change; or

**8.** chromosome-end exclusion leaves insufficient internal sequence for prediction.

Before changing parameters, determine whether the pattern occurs on only one chromosome or repeatedly across the genome. A recurrent pattern on several chromosomes provides stronger justification for a genome-wide adjustment. A single anomalous chromosome may instead reflect an assembly gap, incomplete annotation, unusual chromosome structure, or an unreliable feature track.

# S1.5 Troubleshooting an unusually narrow prediction

An unusually narrow candidate should first be inspected to determine whether it represents:

**•** a genuine compact regional signal;

**•** one isolated TE-rich window;

**•** one isolated tandem-repeat array;

**•** the edge of a broader gene-free region;

**•** a chromosome-terminal repeat signal;

**•** a local annotation gap; or

**•** a narrow peak competing with a broader internal regional maximum.

A short candidate should be retained when it occurs within a coherent accumulated-score maximum and is supported by several neighboring windows and complementary features.

Adjustment is appropriate when the selected narrow interval is dominated by one isolated feature while another internal region shows broader and more consistent support. The example weight changes below are controlled genome-wide diagnostic adjustments for resolving a specific output pattern. They are distinct from the predefined cross-genome feature-weight configurations evaluated in the sensitivity analysis summarized in Table S3.

## S1.5.1 Narrow candidate dominated by TE coverage

If one isolated TE-rich locus dominates the score, reduce the TE weight while retaining all other settings. Suitable diagnostic alternatives include:

**4/8/1/1/1/1 → 4/4/1/1/1/1**

**or:**

**4/8/1/1/1/1 → 4/3/1/1/1/1**

The alternative configuration must be applied to the complete genome. Compare the location and regional support of every chromosome-level candidate before and after the change.

The reduced TE weight should be retained only when it removes an isolated TE-driven candidate, allows a coherent regional signal to become dominant, and preserves the locations of the other well-supported candidates.

## S1.5.2 Narrow candidate dominated by tandem repeats

If the selected candidate corresponds primarily to one isolated tandem-repeat array, test a moderate reduction in the tandem-repeat weight:

**4/8/1/1/1/1 → 2/8/1/1/1/1**

A more balanced repeat configuration may also be evaluated when both tandem-repeat density and TE coverage dominate isolated loci:

**4/8/1/1/1/1 → 2/2/1/1/1/1**

The alternative should not be retained simply because it produces a longer interval. It should be retained only when it selects a more coherent internal regional signal and does not destabilize the other chromosome-level predictions.

## S1.5.3 Broader internal region supported by GC depletion

If the common configuration selects a narrow or competing region but the plot shows a broader internal gene-free region with strong GC depletion and support from other features, increase the GC-depletion weight incrementally.

**The first adjustment should normally be:**

**4/8/1/1/1/1 → 4/8/1/1/1/2**

If the same broad internal region remains consistently supported but is still underweighted, higher GC-depletion values may be tested. Each configuration must be applied across the complete genome while all other settings remain unchanged.

The purpose of increasing the GC-depletion weight is not to make the predicted interval longer. The adjustment is justified only when the plot indicates that a broad, internally coherent, GC-depleted region is being outscored by a narrow or compositionally misleading alternative.

# S1.6 Troubleshooting multiple competing regions

When two or more distant regions have similar accumulated regional scores, compare:

**•** the width and continuity of each regional maximum;

**•** the number of neighboring elevated individual-window scores;

**•** whether each region occurs within a continuous gene-free interval;

**•** the number of features supporting each region;

**•** whether one candidate is close to a chromosome end;

**•** whether one candidate corresponds to an isolated TE or tandem-repeat locus;

**•** whether coverage or methylation anomalies coincide with assembly gaps; and

**•** whether one region remains selected under a moderate alternative configuration.

Only one feature or parameter class should initially be changed. For example:

**•** reduce the TE weight when one isolated TE-rich region dominates;

**•** reduce the tandem-repeat weight when one tandem-repeat array dominates;

**•** increase the GC-depletion weight when a broader internal GC-poor region is consistently visible;

**•** adjust methylation or coverage weights only when the corresponding input track is high quality and shows a broad regional pattern rather than isolated technical spikes.

A prediction is considered stable when the same general genomic region is retained under the common configuration and at least one biologically reasonable alternative configuration. Small boundary changes are expected because candidate boundaries depend on the accumulated regional-score profile and the extent of the corresponding gene-free region.

If two distant regions remain similarly supported and the selected location changes substantially under small genome-wide parameter changes, the chromosome should be reported as ambiguous rather than assigned a chromosome-specific configuration.

# S1.7 Troubleshooting no returned candidate

When CentroFinder does not return a candidate for a chromosome, first determine whether the chromosome contains valid searchable sequence after chromosome-end exclusion.

Check:

**•** whether twice the applied end-exclusion distance equals or exceeds the chromosome length;

**•** whether the chromosome was skipped with a warning;

**•** whether the remaining internal sequence contains a continuous gene-free region;

**•** whether one or more feature files lack values for that chromosome;

**•** whether chromosome identifiers are consistent among all input files;

**•** whether the chromosome length matches the coordinates in the annotation files;

**•** whether extensive assembly gaps are present; and

**•** whether the sequence was incorrectly classified as mitochondrial, contaminant, or unplaced sequence.

If an excessive chromosome-end exclusion leaves no internal search space, reduce the exclusion distance genome-wide to a value that retains sufficient searchable sequence. Do not reduce the exclusion distance for only one chromosome.

If valid input data and sufficient internal sequence are present but no coherent accumulated-score maximum is produced, the chromosome should be reported as having no confident prediction rather than repeatedly adjusted until an interval is forced.

# S1.8 Troubleshooting recurrent chromosome-terminal predictions

Chromosome-terminal regions may contain strong TE, tandem-repeat, gene-depletion, or GC-depletion signals that compete with internal centromeric candidates. The default chromosome-end exclusion is intended to prevent these terminal signals from dominating the analysis.

**A single terminal prediction should first be examined for:**

**•** chromosome truncation or misassembly;

**•** an incorrect chromosome length;

**•** incomplete terminal gene annotation;

**•** telomeric or subtelomeric repeat arrays;

**•** assembly gaps;

**•** the presence of a broader internal accumulated-score maximum.

**A genome-wide increase in chromosome-end exclusion is justified when:**

**•** terminal or near-terminal candidates occur on several chromosomes;

**•** the terminal candidates are dominated by repeat-rich or gene-poor sequence;

**•** broader internal regional-score maxima remain visible;

**•** increasing the exclusion resolves the same pattern across multiple chromosomes; and

**•** the larger exclusion does not remove most of the searchable sequence from shorter chromosomes.

Test the exclusion distance stepwise while keeping the feature weights fixed. For example:

**100 kb → 250 kb → 350 kb**

The revised exclusion distance should be applied consistently to all sequences meeting the large-sequence cutoff.

If the combined left and right exclusions leave no valid internal search space, the chromosome should be skipped with a warning rather than assigned a forced candidate.

**Example: *Fusarium oxysporum f. sp. vasinfectum* ME23**

The common 4/8/1/1/1/1 configuration with the 100-kb chromosome-end exclusion produced terminal or near-terminal candidates on three chromosomes:

**•** CP130300.1: 292,000–316,000 bp;

**•** CP130306.1: 144,000–173,000 bp; and

**•** CP130308.1: 260,000–263,000 bp.

The CP130308.1 prediction was only 3,001 bp long. Inspection of the diagnostic plots showed strong terminal score maxima together with broader internal accumulated-score peaks.

Because the same pattern occurred on multiple chromosomes, it was treated as a genome-wide chromosome-end competition problem rather than as separate chromosome-specific feature-weight problems.

The large-sequence chromosome-end exclusion was increased from 100 to 350 kb across the genome while the 4/8/1/1/1/1 feature weights and all other parameters were retained. The revised internal candidates were:

**•** CP130300.1: 2,040,000–2,078,000 bp;

**•** CP130306.1: 1,684,000–1,716,000 bp; and

**•** CP130308.1: 1,043,000–1,081,000 bp.

The remaining chromosome-level predictions retained their original locations. The adjustment was therefore retained because it removed recurrent terminal competition without destabilizing otherwise coherent predictions.

# S1.9 Examples of genome-wide feature-weight adjustment

## S1.9.1 *Magnaporthe oryzae* Guy11

The common 4/8/1/1/1/1 configuration produced coherent predictions on six chromosomes. On Chr2, however, the selected interval at 2,362,000–2,402,000 bp did not correspond to the broader internal region supported by the GC-depletion profile and the other regional features.

The GC-depletion weight was increased from 1 to 2 across all seven chromosomes:

**4/8/1/1/1/1 → 4/8/1/1/1/2**

The Chr2 prediction changed to 314,000–411,000 bp, while the six previously coherent chromosome-level predictions retained their locations. The revised Chr2 interval also overlapped the experimentally defined centromere.

This example illustrates the general adjustment procedure: the diagnostic plot identified an underweighted broad internal feature pattern, one weight was changed genome-wide, and the change was retained because it resolved the problematic chromosome without destabilizing the remainder of the genome. The availability of experimentally defined coordinates provided an additional benchmark but was not required for identifying the original inconsistency in the plot.

## S1.9.2 *Aspergillus luchuensis* IFO 4308

The common configuration produced a 1,001-bp candidate at 726,000–727,000 bp on AP024428.1. The diagnostic plot showed that this narrow candidate was less regionally coherent than a broader internal region supported by GC depletion and complementary features.

**The GC-depletion weight was increased genome-wide from 1 to 5:**

**4/8/1/1/1/1 → 4/8/1/1/1/5**

The AP024428.1 candidate changed to 1,908,000–2,007,000 bp, while the predictions on the remaining chromosomes retained their locations.

The revised interval was not selected merely because it was longer. The adjustment was retained because the new candidate corresponded to a broader, internally coherent regional-score maximum and did not disrupt the other chromosome-level predictions.

## S1.9.3 *Penicillium psychrofluorescens* KMR99

The common configuration produced a 5,001-bp candidate at 280,000–285,000 bp on OZ078162.1. Inspection of the diagnostic plot identified a broader internal region with stronger regional support from GC depletion and complementary features.

Increasing the GC-depletion weight from 1 to 2 across all chromosomes changed the prediction to 2,298,000–2,328,000 bp:

**4/8/1/1/1/1 → 4/8/1/1/1/2**

The candidates on the other chromosomes retained their locations. The adjusted configuration was therefore retained.

These examples do not indicate that GC depletion should routinely be up-weighted. Most analyzed genomes retained the common 4/8/1/1/1/1 configuration. GC-depletion adjustment should be considered only when the diagnostic plot shows a broad, coherent internal GC-poor region that is underrepresented by the default weighting.

# S1.10 Secondary adjustment of window size

Window-size adjustment should be considered only after input quality, feature weighting, and chromosome-end exclusion have been evaluated.

The 1-kb window should normally be retained. A different window size may be tested when:

**•** the individual-window score profile is excessively noisy;

**•** multiple immediately adjacent narrow peaks appear to form one broader region;

**•** candidate boundaries repeatedly appear limited by the 1-kb resolution; or

**•** the chromosome architecture produces a regional signal that is fragmented across many small windows.

A smaller window may improve boundary resolution but may increase noise. A larger window may smooth fragmented regional signals but may reduce positional resolution.

Any alternative window size must be applied to the complete genome while all feature weights and chromosome-end settings remain fixed. The alternative should be retained only if it improves the coherence and stability of the regional signal across the genome, not merely because it produces longer or shorter intervals.

# S1.11 Missing or unreliable feature tracks

Before setting any feature weight to zero, confirm that the missing signal is not caused by:

**•** an incorrect input path;

**•** inconsistent chromosome identifiers;

**•** an empty annotation file;

**•** an unsupported file format;

**•** missing chromosomes in the input data;

**•** failed long-read mapping; or

**•** failed methylation calling.

If a feature is genuinely unavailable, the corresponding weight may be set to zero as a reduced-feature analysis. The reduced-feature configuration must be applied to the complete genome, and the remaining weights should initially be retained.

**For example:**

**4/8/1/1/1/1 → 4/8/1/0/1/1**

when CpG methylation data are unavailable.

A reduced-feature analysis is not directly equivalent to the complete six-feature model, and candidate stability should therefore be evaluated carefully, particularly when one of the dominant repeat-associated features is unavailable.

A feature track containing strong technical spikes, extensive missing regions, or alignment artifacts should not be up-weighted. Coverage and methylation weights should be increased only after confirming the quality of the underlying reads, alignments, and chromosome-wide profiles.

# S1.12 Criteria for retaining an adjusted configuration

**A modified configuration may be retained when:**

**•** the original problem is clearly visible in the diagnostic plots;

**•** the parameter change directly addresses that problem;

**•** only one parameter class is changed at a time;

**•** the revised setting is applied across the complete genome;

**•** the problematic chromosome or chromosomes acquire a coherent internal candidate;

**•** most previously coherent candidates retain their locations;

**•** no new terminal, isolated, or competing predictions are created;

**•** the revised candidate is supported by neighboring windows and complementary features; and

**•** the result can be reproduced using the same settings.

**An adjustment should be rejected when it:**

**•** moves many previously stable candidates to unrelated regions;

**•** requires different weights for individual chromosomes;

**•** produces new competing candidates;

**•** depends almost entirely on one potentially artifactual feature;

**•** removes most of the searchable sequence;

**•** forces a candidate where no coherent regional maximum exists; or

**•** is justified only because it produces a preferred interval length.

Where experimentally defined centromere coordinates are available, overlap with those coordinates may provide additional support for the selected configuration. However, agreement with experimental coordinates should not be achieved through chromosome-specific tuning.

# S1.13 Summary workflow

**1.** run CentroFinder using the common 4/8/1/1/1/1 settings;

**2.** inspect the diagnostic plot for every chromosome;

**3.** verify the input files before changing parameters;

**4.** retain the default when one coherent internal candidate is produced;

**5.** examine unusually narrow intervals for isolated feature dominance or a competing broader region;

**6.** modify only the parameter directly related to the identified problem and apply the change genome-wide;

**7.** increase chromosome-end exclusion when recurrent terminal competition occurs;

**8.** consider window-size adjustment only after the other causes have been evaluated;

**9.** compare all chromosome-level outputs before and after each change;

**10.** retain an adjustment only when it resolves the identified problem and preserves otherwise coherent candidates; and

**11.** retain unresolved or ambiguous chromosomes as such rather than applying chromosome-specific tuning.

**Use of generative AI**

OpenAI ChatGPT was used to assist with language polishing, readability improvement, consistency checking, and preparation of the supplementary benchmarking visualization. All outputs were reviewed and verified by the authors.
