# Supplementary material for: CentroFinder: a multi-feature framework for de novo prediction of fungal regional centromeres
Source: Bioinform Adv. 2026 Sep 12;6(1):vbag270. doi: 10.1093/bioadv/vbag270 (PMC13682844; doi:10.1093/bioadv/vbag270)
Supplement: Supplementary file 1 [file vbag270_supplementary_data.zip › Figure_S1_Finalized.pdf]

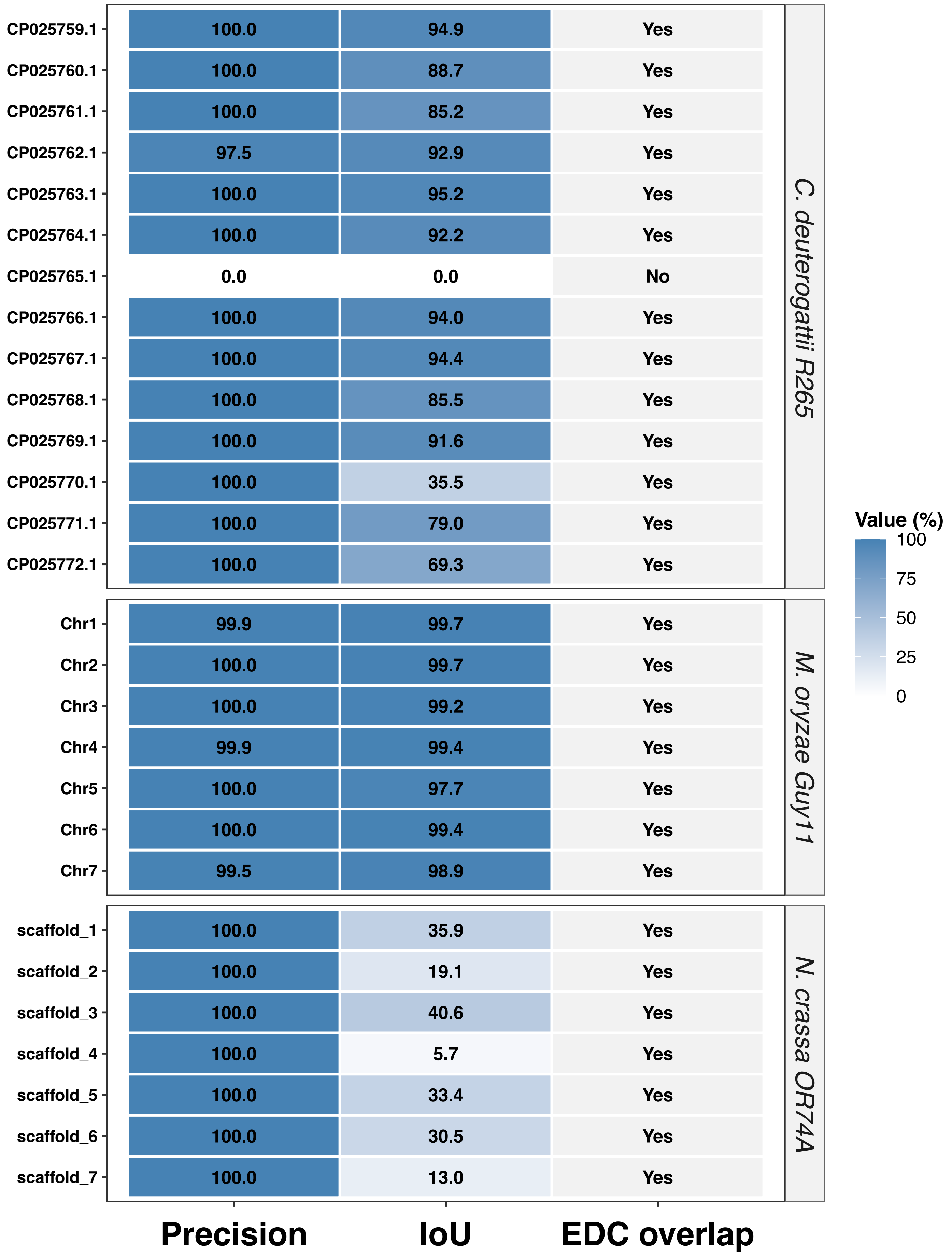
CP025759.1
100.0
94.9
Yes
CP025760.1
100.0
88.7
Yes
CP025761.1
100.0
85.2
Yes
CP025762.1
97.5
92.9
Yes
CP025763.1
100.0
95.2
Yes
CP025764.1
100.0
92.2
Yes
CP025765.1
0.0
0.0
No
CP025766.1
100.0
94.0
Yes
CP025767.1
100.0
94.4
Yes
CP025768.1
100.0
85.5
Yes
CP025769.1
100.0
91.6
Yes
CP025770.1
100.0
35.5
Yes
CP025771.1
100.0
79.0
Yes
CP025772.1
100.0
69.3
Yes
C. deuterogattii R265
Chr1
99.9
99.7
Yes
Chr2
100.0
99.7
Yes
Chr3
100.0
99.2
Yes
Chr4
99.9
99.4
Yes
Chr5
100.0
97.7
Yes
Chr6
100.0
99.4
Yes
Chr7
99.5
98.9
Yes
M. oryzae Guy11
scaffold_1
100.0
35.9
Yes
scaffold_2
100.0
19.1
Yes
scaffold_3
100.0
40.6
Yes
scaffold_4
100.0
5.7
Yes
scaffold_5
100.0
33.4
Yes
scaffold_6
100.0
30.5
Yes
scaffold_7
100.0
13.0
Yes
N. crassa OR74A
Precision
IoU
EDC overlap
Value (%)
100
75
50
25
0

Figure S1

Figure S1. Chromosome-level quantitative benchmarking of CentroFinder against experimentally defined fungal centromeres. CentroFinder predictions were compared with experimentally defined centromeres (EDCs) across 28 chromosomes from *Cryptococcus deuterogattii* R265, *Magnaporthe oryzae* Guy11, and *Neurospora crassa* OR74A. Precision represents the proportion of the predicted interval that overlaps the EDC, whereas intersection-over-union (IoU) represents the overlap between the predicted and experimentally defined intervals divided by the union of the two intervals and therefore measures overall agreement between them. EDC overlap indicates whether the predicted and experimentally defined intervals share at least one base pair. Values are shown for all chromosomes for which experimentally defined centromere coordinates were available. *N. crassa* scaffolds 8 and 9 were excluded from quantitative comparison because experimentally defined centromere coordinates were unavailable. In *N. crassa*, the high precision but lower IoU values reflect CentroFinder predictions that are fully contained within, but narrower than, the broader experimentally defined centromeric regions.
